# Pilot Aquaphotomic Study of the Effects of Audible Sound on Water Molecular Structure

**DOI:** 10.3390/molecules27196332

**Published:** 2022-09-26

**Authors:** Aleksandar Stoilov, Jelena Muncan, Kiyoko Tsuchimoto, Nakanishi Teruyaki, Shogo Shigeoka, Roumiana Tsenkova

**Affiliations:** 1 Yunosato Aquaphotomics Lab, Hashimoto 648-0086, Wakayama, Japan; 2 Aquaphotomics Research Department, Graduate School of Agricultural Science, Kobe University, Kobe 657-8501, Hyogo, Japan

**Keywords:** aquaphotomics, near-infrared spectroscopy, water, light, sound, frequency, perturbation, aquagram, molecular dynamics

## Abstract

Sound affects the medium it propagates through and studies on biological systems have shown various properties arising from this phenomenon. As a compressible media and a “collective mirror”, water is influenced by all internal and external influences, changing its molecular structure accordingly. The water molecular structure and its changes can be observed as a whole by measuring its electromagnetic (EMG) spectrum. Using near-infrared spectroscopy and aquaphotomics, this pilot study aimed to better describe and understand the sound-water interaction. Results on purified and mineral waters reported similar effects from the applied 432 Hz and 440 Hz frequency sound, where significant reduction in spectral variations and increased stability in water were shown after the sound perturbation. In general, the sound rearranged the initial water molecular conformations, changing the samples’ properties by increasing strongly bound, ice-like water and decreasing small water clusters and solvation shells. Even though there was only 8 Hz difference in applied sound frequencies, the change of absorbance at water absorbance bands was specific for each frequency and also water-type-dependent. This also means that sound could be effectively used as a perturbation tool together with spectroscopy to identify the type of bio, or aqueous, samples being tested, as well as to identify and even change water functionality.

## 1. Introduction

Sounds are mechanical waves of pressure that propagate through a transmission medium. Being a wave of pressure, the sound acts as a mechanical stimulus and has an influence on the medium through which it propagates. In physics, sound waves and their properties are widely investigated and generally well-understood, however in life sciences the investigation of sound has not yet received full attention.

There are many records documenting how sound affects biomolecules in water solutions, single-cells and even whole organisms, plants especially [1,2,3,4,5,6,7]. One study showed that audible sound in the form of music (38–689 Hz) was able to affect growth, metabolism and antibiotic susceptibility of prokaryotic as well as eukaryotic microbes [8,9]. A study about the influence of sound on chrysanthemum plants, discovered that sound wave accelerated the synthesis of RNA and soluble protein, indicating that some stress-induced genes might be switched on under sound stimulation. Further, another study showed that survival rate in the conditions of water deprivation is significantly higher in sound-treated Arabidopsis adult plants compared to plants kept in silence [2]. This study revealed significant upregulation of 87 genes, the majority of which are responsible for abiotic stress response, pathogen responses and oxidation reduction processes; interestingly, two genes were involved in the responses to mechanical stimulus.

The effects of sound on biological structures was also investigated in vitro, showing, for example, that crystallization of proteins is sensitive to audible sound and is frequency-dependent [6,10]. Sound, however, has not only been seen to induce changes by pure mechanical means, it has also been shown to have an impact on the signal paths of plants and the nervous system of animals. In a study evaluating the effects of different musical frequencies (432 and 440 Hz) on food intake and body weight in rats, it was discovered that different frequencies affect the neuronal expressions of two peptides involved in regulation of food intake in the rat hypothalamus, which stimulated weight increase (higher weight increase was found for 440 Hz) [11]. Listening to low frequency music (432 Hz) was shown to significantly decrease heart rate in high-blood pressure individuals [12].

Recent studies demonstrated clear evidence on sound-induced liquid vibrations that can lead to the formation of chemically (pH or redox) different spatiotemporal domains in solutions. This approach was later used to specifically align nanofibers in solution utilizing fluid flows generated by audible sound vibrations [13,14].

Regardless of the description of sound effects on different organizational levels, from biomolecules to organisms, the specific process of underlying molecular mechanisms, especially in the case of biological systems, is still poorly understood. Up to very recently, scientists were focusing on pinpointing single biomolecules related to sound influence, without considering the contribution of all components, especially without considering water, which is actually a major component of all biological systems. Since biological systems are water systems, the sound-water interaction is of interest and aquaphotomics science and technology [15,16] offer a non-invasive way to investigate this phenomenon. This discipline uses the water molecular network as a mirror [15] or a sensor [16] to characterize the observed system by monitoring the electromagnetic (EMG) spectral pattern of water under various perturbations. With the help of the aquaphotomics method, this pilot study aims to better understand how sound alters the molecular structure and dynamics of water using music tuned at two frequencies—432 Hz and 440 Hz. 

The frequency 440 Hz was chosen, as it is the audio frequency which serves as a tuning standard for the musical note A4 above middle C, standardized by the International Organization for Standardization (ISO 16) in 1955 [17]. The 432 Hz was chosen because of the increasing number of claims that this frequency is superior to the standard tuning 440 Hz, affecting the body, emotional response, timbre, sound quality, character and tone; while it is actually unclear if it is humanly possible to discern a difference between the two [18,19]. According to existing scientific studies, music tuned at 432 Hz, compared to 440 Hz, can significantly decrease the heart rate and slightly decrease mean respiratory rate values [19]; it results in better cardiovascular benefits including slowing heart rate and it promotes relaxation [12]. It significantly decreases dental anxiety levels [20,21], significantly improves the sleep scores of patients with spinal cord injuries [22] and leads to increased levels of perceived arousal [23]. On the other hand, there are reports suggesting that listening to 440 Hz music can promote a feeling of anxiety, nervousness or aggression [19]. The research study about the effects of sound of different frequencies found that human DNA is sensitive to music, and that in fact by subjecting stem cells to various frequencies, it is possible to modify their natural organic function [24]. The actual mechanism behind all these phenomena is still unknown. The possibility of influencing the human body on a biomolecular level and stimulating, modifying or repairing the functionality of vital biomolecular structures just by using sound, holds tremendous potential. As a first step towards this goal, it is necessary to understand the role that frequency plays in sound effects and provide the information on which the choice of frequency for sound can be performed.

## 2. Results

### 2.1. Reference Measurements

Near-infrared spectroscopy (NIRS) in the range of 900–1700 nm, in parallel with monitoring of several physico–chemical parameters, was used to characterize pure water (hereinafter PW) and a chosen mineral water (hereinafter MW), both measured before and after sound perturbation at the frequencies of 432 Hz and 440 Hz. The mean values and standard deviations of measured electrical conductivity, pH, salinity and temperature of the samples through 10 min of sound are presented in Table 1. pH values were observed to be slightly more resilient to change in samples after the perturbation was applied, where the waters had an average of 0.81% and 0.38% more stable pH, respectively, after being perturbed by the sound with 432 Hz and 440 Hz frequencies. The difference in variance between samples that were perturbed and the control ones was not found to be statically significant (data not shown), however, statistical significance is not the same as practical importance [25]. Increased changes in values were seen for electrical conductivity of more than 18% on average after sound. These parameters are hard to measure precisely for waters, especially for PW. As soon as the vial is opened there is an exchange with the atmosphere of the environment; some evaporation may occur, gases from the atmosphere, such as CO_2_ and O_2_, may influence the resulting measurements of pH and electroconductivity. The changes in pH are, for example, very small, and due to the inherent probe instability [26] may not reflect accurate values. This all points out the difficulties of reference methods usually used to describe the effects of perturbations that may be very delicate, but nevertheless genuine and consistent, which requires devising other means and more controlled measurements which can completely eliminate possible influences of the environment. There is also a possibility that these physico-chemical parameters may simply be inadequate to capture all of the information relevant for the thorough description of water as a complex system and its dynamics in response to some perturbations [27].

However, despite this, the current results pointed to some degree of stability of water properties in the presence of sound. One possible way of interpretation this is that sound as a mechanical wave-inducing constant perturbation on the samples, reduces the susceptibility to influences from other factors. This phenomenon is further investigated in the following analyses.

The mean temperature values showed the tendency to decrease after the sound application, irrespective of the water type or the frequency of the applied sound, while the salinity did not change at all, evidencing that sound does not change the chemical components in the water. The change in temperature might be a result of absorbed heat generated by the sound perturbation. Previous works have shown that at least part of the audible sound energy is transformed into heat that can be absorbed by water, and spent on the reorganization of water molecular structure, causing the change in its temperature [6,10].

### 2.2. Aquaphotomic Spectral Analysis

#### 2.2.1. Initial Exploration of Spectra: Raw Spectra of Water, Preprocessed Spectra and Difference Spectra

**Figure 1:**
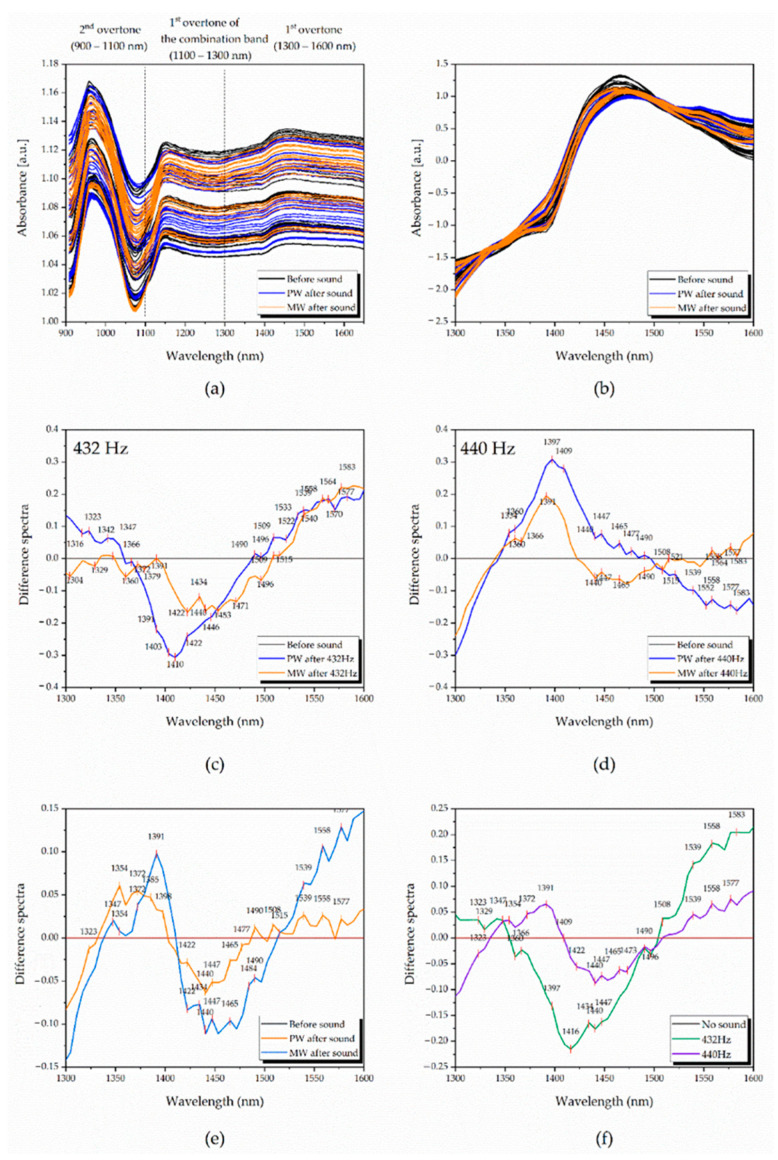
Near-infrared spectra of tested pure and mineral waters and difference spectra analyses (DSA) on defined datasets, preprocessed with SNV: (**a**) Raw spectra, separated into the water overtones; (**b**) raw spectra at the 1st water overtone (1300–1600 nm) transformed with SNV and smoothed with Savitzky–Golay filter using 2nd order polynomial (9 points), showing decreased baseline offset; (**c**) difference spectra analysis of 432 Hz data, with specific spectral patterns for each water; (**d**) difference spectra analysis of 440 Hz data with different spectral patterns than those at 432 Hz; (**e**) difference spectra analysis of averaged water types, displaying similarities in the spectral curves and differences in regions related to small water clusters and water ions; (**f**) difference spectra analysis of averaged frequencies, showing how the gap of 8 Hz can affect water differently.

The raw water absorbance spectra generated during the experiments are presented in Figure 1a. The spectra show absorbance higher than OD = 1 a.u. and are rather flat except for the large, broad peak at lower wavelengths.

However, on closer inspection, several spectral features could be observed: large absorbance band centered around 957–963 nm corresponds to the 2nd overtone of water OH stretching vibrations, smaller band corresponding to the overtone of the combination band of free water molecules can be detected around 1143–1155 nm, and a broad peak corresponding to the 1st water overtone of water stretching vibrations can be seen located in the region 1300–1600 nm. The spectral profiles of PW show a generally higher spectral profile compared to the MW. This baseline shift is generated partially from the unfixed environmental conditions (temperature, humidity, water layer on the surface of the sample’s vials, etc.), that may influence scattering of light and lead to differences in the real pathlength of light, but the higher spectral profile of PW can also be due to the higher actual water content since MW contain lots of minerals. When applying SNV preprocessing on the raw data, the baseline effects were removed (Figure 1b). Calculating difference spectra (Figure 1c–f) yielded more information on the effects of sound. This method requires the subtraction of a sample spectrum, usually of the control group (in this case the waters before sound perturbation), from the spectra of the other samples (waters after sound perturbation). The calculated difference spectra presented in Figure 1c,d, respectively, display 432 Hz and 440 Hz datasets with averaged waters’ spectra after subtraction of averaged spectrum of the control.

In the subtracted spectra, three groups of common peaks were observed that correspond to well-known water absorbance bands: water confined in a local field of ions or so-called trapped water (1391–1397 nm) [28], water solvation shells and proton hydrates (1347–1385 nm) [15,29,30,31] and strongly bound water (1515–1590 nm) [15,32].

After averaging the whole spectra of PW and MW (Figure 1e), and subtracting their controls, a common increase in light absorption was observed at 1335–1403 nm and 1515–1600 nm, regions related to free, trapped and strongly-bounded water, respectively, while the absorbance in the in-between region (~1416–1490 nm) showed a decrease after the sound perturbation. The absorbance in the spectral region of absorbance of small water clusters with one hydrogen bond (1440–1444 nm), two hydrogen bonds (1462–1468 nm), three hydrogen bonds (1476–1482 nm) and four hydrogen bonds (1488–1494 nm) [15] showed differences between the two examined waters, but the common feature for both was decreased light absorption in general. The absorbance at absorbance bands corresponding to water solvation shells (1360–1366 nm and 1380–1388 nm) [15], hydronium ions (1434–1438 nm) [15], protonated and hydroxylated water clusters (1564 nm, 1583 nm and 1589 nm) [29,30,31], as well as water superoxide ions [31], increased after the sound perturbations. Although PW and MW are both waters and exhibited similar spectral curvatures, a contrast emerged describing unique characteristics for each sample type. As a mineral water, having minerals, after sound stimulus, MW was seen with much higher absorption at the bands related to water ions (water solvation shells (1380–1388 nm) [15] and trapped water (1391–1397 nm) [32]).

When calculating average spectra according to the applied sound frequency (Figure 1f), water samples after 432 Hz sound stimulus were characterized by a higher increase in ionic water species, while displaying less free water molecules and small water clusters compared to the molecular structure of water after 440 Hz sound stimulus. This finding suggests that a particular tuning even with only 8 Hz difference could generate different effects, which by themselves are dependent on the type of media/water being perturbed (sample-dependent) and are related to respective changes of water functionalities [15,16,33,34].

#### 2.2.2. Exploratory Analysis-Principal Component Analysis (PCA) 

**Figure 2:**
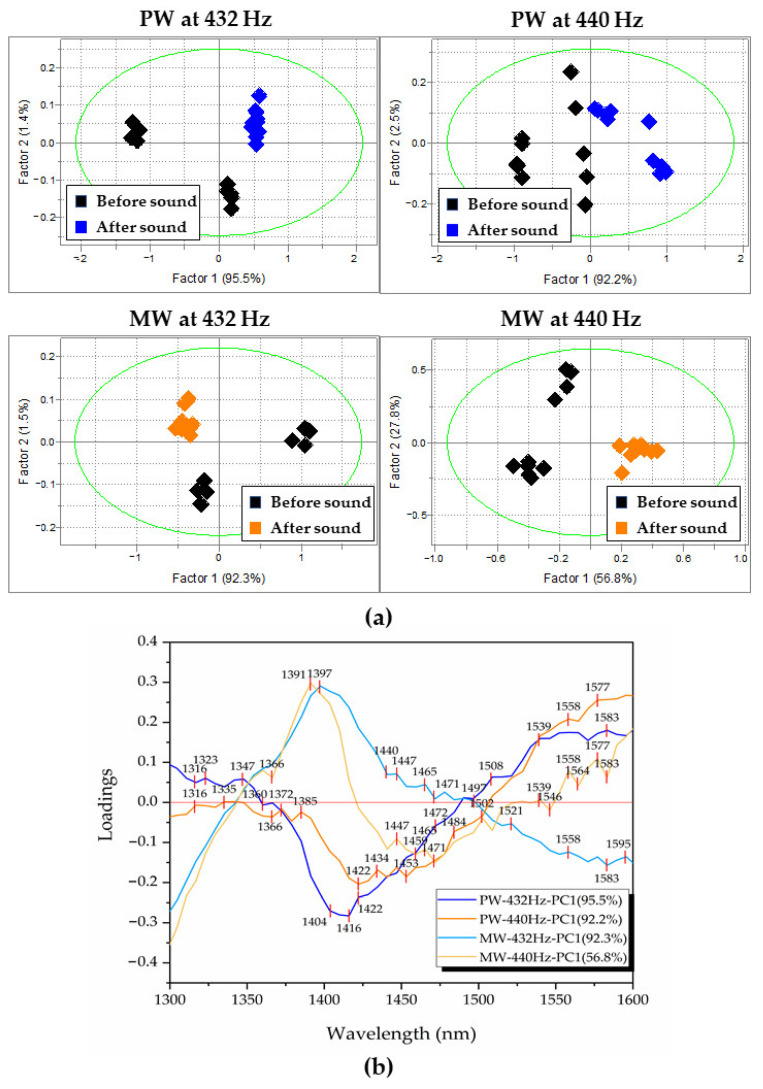
PCA scores and loadings separating samples before and after sound: (**a**) Scores of 1st and 2nd principal components for the 4 datasets; (**b**) loadings of 1st principal components for the 4 datasets, separating before from after samples (it should be noted that the sign of PC loadings is assigned arbitrarily, and due to this, the loading of PC1 for MW at 432 Hz was multiplied by −1).

In order to better understand the effects of sound and confirm the initial findings, further analysis was performed—PCA (principal component analysis) with mean-centering and SNV preprocessing applied on spectral data. The experimental dataset was split into four datasets, according to the water type and sound frequency (PW–432 Hz, PW–440 Hz, MW–432 Hz, MW–440 Hz) and then PCA was applied to explore if there are any patterns or groupings in the spectra that can be related to the applied sound perturbation (Figure 2). The score plot of PC1 vs. PC2 from each PCA analysis are presented in Figure 2a and show repeatable patterns where generally scores of waters after sound perturbation showed diminished dispersion (inter-class variation) in the PC1–PC2 defined space. This implied that the sound-perturbed waters were more stable, becoming less prone to environmental influences, such as temperature, humidity, light, etc., which is consistent with the previous findings based on water parameters. The separation of the scores before and after sound perturbation was also a common property for all datasets and could be observed in the score space of PC1 vs. PC2. The loadings of PC1 and PC2 are given in Figure 2b and show that the absorbance bands that explain this separation of scores according to the sound perturbation are the same wavelengths already observed in the analysis of the subtracted spectra. The shape of PCA loadings was similar to the spectral profiles of subtracted spectra shown in Figure 1c,d, and again, but here in PC1 loadings, MW showed smaller differences compared to the PW for both 432 Hz and 440 Hz frequencies. On the other hand, PW being more receptive of changes, displayed opposing peaks in the 1366–1490 nm region, with direction shift different for the two frequencies.

Looking at the score plots of PCA analysis of PW (Figure 2a) it can be observed that in both cases, after the sound perturbation, the scores of PW are located in the positive part of PC1. The loading of PC1 for the dataset of sound perturbation by 432 Hz, shows the most prominent spectral feature as a negative peak at 1416 nm, which means that absorbance at this band increases under the sound influence.

For the 440 Hz dataset, the most prominent feature is the peak at 1397 nm, which can be interpreted as an increase in absorbance at this band under the influence of sound. The absorbance band at 1416 nm can be assigned to the free water, but it is more likely that this band can be attributed to so-called hydration water molecules [15], while 1397 nm is assigned to quasi-free water molecules, the single water molecules trapped in the local field of ions [28]. Similarly, for the score plots of PCA analysis of MW, the scores in the case of the 432 Hz dataset are mostly in the negative part of PC1, while in the case of 440 Hz they are in the positive part. In this case, in order to be able to compare the shape and sign of all loadings (Figure 2b), due to the arbitrary assignment of loadings in PCA, the loading vector of PC1 in the case of the MW dataset for 432 Hz frequency had to be multiplied by −1; therefore, the interpretation of the meaning of the scores of the MW dataset for 432 Hz is actually reversed. Having this in mind, it can be observed from the loadings plot (Figure 2b) that most distinctive features are a large negative peak at 1422 nm for the 432 Hz dataset PC1 loading, and a positive peak at 1391 nm in the case of the 440 Hz dataset PC1 loading. In the first case, this result means that sound of 432 Hz leads to an increase of absorbance at 1391 nm (trapped water band), while sound of 440 Hz leads to an increase in absorbance at 1422 nm (hydration band). From this, it can be concluded that the sound of 432 Hz frequency shows common effect for both waters, the increased absorbance of trapped water molecules, while 440 Hz sound increases the absorbance of hydration water. In both cases, this increase comes at the expense of reorganization of hydrogen-bonded water (absorbance bands at wavelengths longer than 1440 nm).

There is also, one more interesting observation. Looking into the percentage of explained variation, it can be seen that PC1 for all datasets explains more than 90% of variation in the spectral data, except in the case of the MW-440 Hz dataset, where PC1 explains only 56.8%, which means that there are additional spectral pattern variations specific for 440 Hz with high variations. 

**Figure 3:**
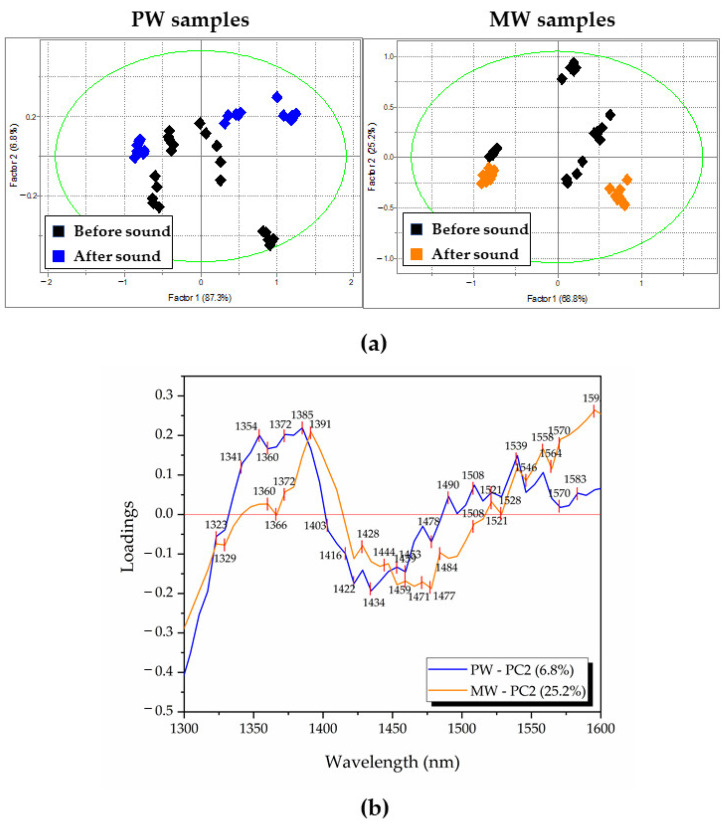
Score plots of PCA analysis of raw spectra preprocessed with SNV for separate PW and MW datasets: (**a**) Score plots for PW data (**left**) and MW (**right**); (**b**) PCA loadings describing influence of sound at PC2 for both waters (positive peaks are related to samples after sound perturbation).

Next, PCA was repeated but this time on two datasets, separated into water type (either only PW or MW) in order to find out what the influence of sound is on waters in general (Figure 3).

In the cases of both waters, the PCA score plots revealed the pattern of separation of scores in groups of “before sound” and “after sound” along the PC2 axis (Figure 3a), whereas the scores corresponding to the “after sound” group were located only on one side of PC2. For the PW dataset, the variance explained by PC2 was 6.8%, while in the case of the MW dataset, a higher percentage of variance (25.2%) was described by the same factor (Figure 3b). Looking at the loadings of PC2 for both PW and MW PCA analysis, familiar absorbance bands similar to the ones from Figure 2b can be seen. This further showed the importance of the already observed bands, as the ones where sound influences the absorbance of water, more precisely, influences particular water molecular structures, especially trapped water (1391–1403 nm) and hydration water (1416–1422 nm). In this analysis, looking at the loadings of PC2, the effects of sound can also be better observed at the region of hydrogen bonded water (1440–1496 nm) where it seems that the small water clusters with 1–4 hydrogen bonds are affected, also, by the sound influence, as well as the strongly bound water (absorbance bands at wavelengths longer than 1508 nm, in particular 1539 nm and 1558 nm).

#### 2.2.3. Discriminating Analysis—Soft Independent Modeling of Class Analogies (SIMCA)

Further, classification analysis, SIMCA, was performed on the already SNV-preprocessed data with 5% significance (95% confidence interval). Three different groups of classes were assigned, such as water type (Class1: PW/Class2: MW), frequency (Class1: 432 Hz/Class2: 440 Hz) and sound perturbation (Class1: before sound perturbation/Class2: after sound perturbation) were performed and their distinction from one another was investigated separately.

First, differentiation between PW and MW was performed on four datasets (waters before 432 Hz, after 432 Hz, before 440 Hz and after 440 Hz) with 100% classification accuracy. It was observed that after the sound the Mahalanobis distances (interclass distances) between the waters decreased by 52% for 432 Hz and 46% for 440 Hz. This pointed out the waters displaying similar properties when stimulated by sound, a tendency seen in previous analysis.

**Figure 4:**
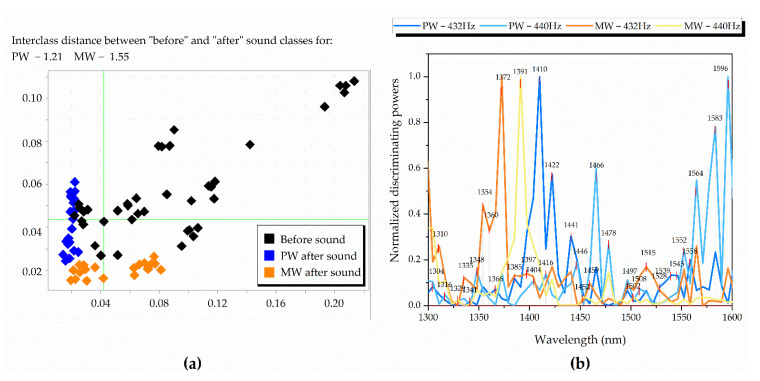
SIMCA classification of waters before and after sound perturbation: (**a**) Cooman’s plot with class distances between PW before sound, PW after sound, MW before sound and MW after sound; (**b**) discriminating powers comparison with applied feature scaling (normalized values from 0 to 1) between PW at 432 Hz, PW at 440 Hz, MW at 432 Hz and MW at 440 Hz, displaying prominent bands for distinguishing the sound states.

Second, the SIMCA analysis performed with the aim of discriminating the samples before and after sound perturbation showed that the classes are different with an interclass distance of 0.76. When the datasets were separated according to the water type, the discrimination accuracy was 98.75% (Figure 4a), but the interclass distances were larger compared to the previous analysis when the waters were put into the same dataset. The values of interclass distances were 1.21 for PW and 1.55 for MW. When the datasets were split into four according to the water type and sound application, and SIMCA analysis was performed once again, the largest value of interclass distance was observed and it was 3.72 between MW before sound and PW after sound. As can be seen from the Cooman’s plot in Figure 4a, the spreading of the scores of samples before sound was more pronounced compared to the scores corresponding to samples after sound perturbation. In order to quantify and compare this “spreading”, the individual PCA models created by SIMCA for each dataset were examined, and the differences between the scores of the same samples were explained in each case by PC2. The percentage of explained variance by PC2 was 1.45% and 1.28%, respectively for PW and MW before sound, decreasing to 0.72% and 0.55% for the respective waters after sound. This was again consistent with previous analyses and pointed towards the influence of sound that can be described as “equalizing”, i.e., reducing the influences of other factors present in the experiment, thus that the measured spectra of the waters showed lower variation among measured replicates. 

In the last step, SIMCA was performed on the same four datasets used in PCA analysis (PW at 432 Hz, PW at 440 Hz, MW at 432 Hz and MW at 440 Hz) to explore the differences before and after sound. The generated discriminating powers show the variables with the highest contribution to the separation of the classes (before sound/after sound). Due to these discriminating powers having different magnitudes unique for the analyzed group of spectra, they were adjusted using feature scaling (unity-based normalization that brings all values into the range of 0–1) for easier comparison, where their recalculated normalized values are presented in Figure 4b. Similar to difference spectra and PCA loadings, the water absorbance bands (WABs) important for discrimination between “before sound” and “after sound” groups, were shown to be dependent on the sample type and located in the same wavelength regions. When the discriminating powers’ prominent peaks (marked with arrows in Figure 4b) were summarized for comparison (Table 2), there was a repeating pattern of variables that were important for discrimination between before and after sound perturbation, and included the following absorbance bands: asymmetric stretching vibrations (1335–1347 nm) [15], H_2_O symmetric stretching proton hydrates H+·(H_2_O)_4_ (1372 nm) [29,30], trapped and free water molecules (1391–1409 nm), water molecules with four hydrogen bonds (1490–1496 nm) [15] and protonated water pentamer (1552–1564 nm) [35]. These water bands were consistent through the four datasets, pointing to a similar effect generated from the perturbation by sound. Small water clusters with three and less hydrogen bonds were also repeatedly seen to be prominent (1440–1477 nm), together with several other wavelength coordinates related to water solvation shells and hydroxylated water clusters (1360–1366 nm) [36,37], hydration band (1422 nm), proton hydrates at 1329–1335 nm [29,35] and at 1583–1589 nm [29].

#### 2.2.4. Aquagrams

**Figure 5:**
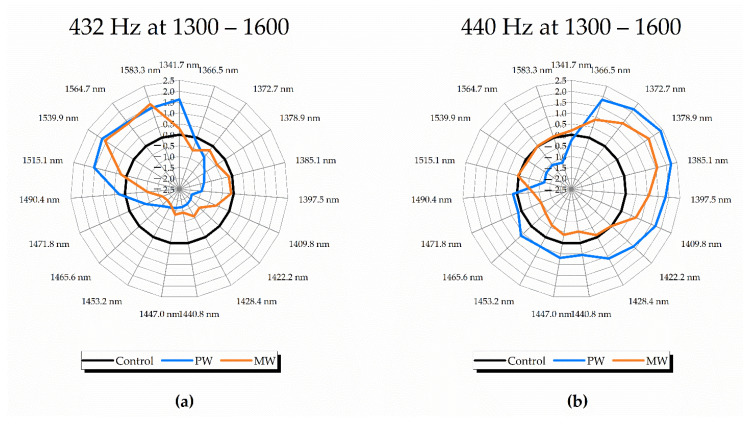
Aquagrams on waters, using prominent WABs from previous analyses, illustrating consistent WASPs at: (**a**) 432 Hz PW and MW at 1300–1600 nm with consistent WASP illustrated as increase in absorbance bands related to aqueous protons and strongly-bounded water; (**b**) 440 Hz PW and MW at 1300–1600 nm with a differently-consistent WASP characterized by higher concentration of water solvation shells, trapped and free water molecules.

**Figure 6:**
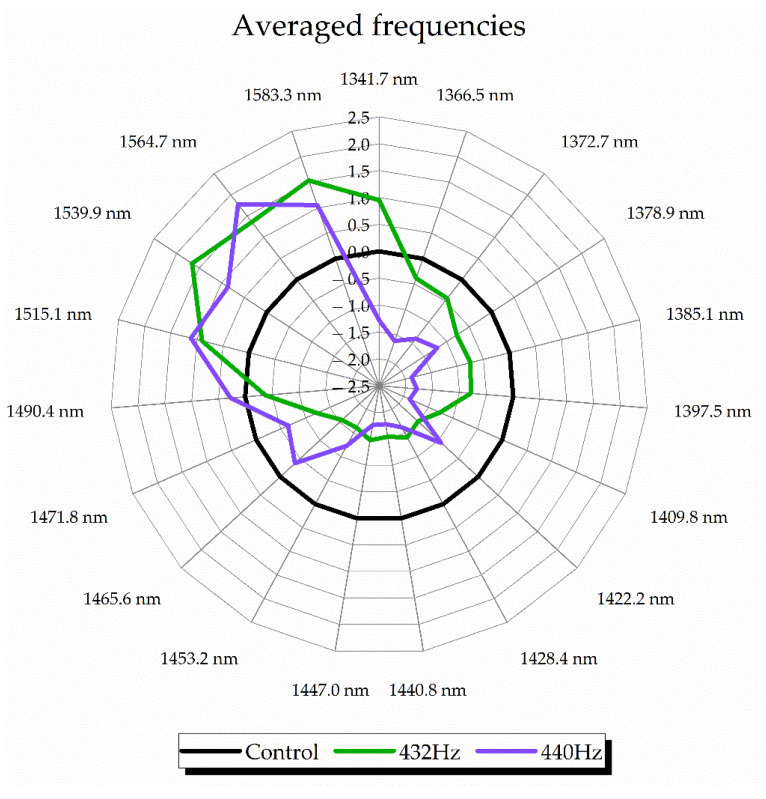
Aquagrams showing average WASPs of water perturbed by sound with 432 Hz and 440 Hz frequencies.

As an important visualization tool of aquaphotomics, aquagrams [15,34,38]—radial graphs presenting the WASP (water spectral pattern) of samples at chosen WABs (water absorbance bands)—were prepared in several ways to emphasize different aspects of the investigation (Figure 5 and Figure 6).

In the first step, spectral absorbance values were standardized using the mean and standard deviation for each wavelength, followed by averaging of PW and MW separately for samples corresponding to before and after sound perturbation. This process was performed for datasets obtained using different sound frequencies, 432 Hz and 440 Hz, in order to present in the aquagram, what the difference in effects of sound is depending on the applied frequency. This result is presented in Figure 5 and distinction between the frequencies was observed at regions related to “ice-like” (strongly-bonded) and “vapor-like” (less bonded) water, respectively [33]. 

Even though the absorbance in the region of small water clusters (1434–1492 nm) decreased after sound perturbation, some specific bands corresponding to the absorbance of water dimer (1440 nm), trimer (1465 nm), tetramer (1472 nm) and pentamer (1490 nm), were still prominent in the case of 440 Hz, but only for the PW. In this case, absorbance was also high at the hydration water band (1422–1428 nm). One more difference between frequencies was observed at the band of proton hydrates (1342 nm), where in the case of 432 Hz, the absorbance was very high, in contrast to the 440 Hz spectral pattern. For 432 Hz samples (Figure 5a), both PW and MW waters shared similar spectral patterns in the 1546–1563 nm region, where the absorbance of hydrogen bonded water and protonated water clusters was increased, while intermediate water species, such as water trimer (1464 nm) and water tetramer (1474 nm) [15], showed tendency of decreased absorbance. Water solvation shells (1364 nm, 1387 nm) and trapped water molecules (1398 nm) did not show changes in MW at that frequency, possibly due to the presence of minerals resulting in the stability of these water species. For PW, proton hydrates (1344 nm) and strongly bound water (1518–1563 nm) were seen to drastically increase, suggesting stronger rearrangements in response to sound. These characteristics, however, were unique for the 432 Hz frequency. At 440 Hz, PW displayed contrasting properties, with a decrease in strongly bound water, while increasing all small water clusters and “vapor-like” water structures (1344–1410 nm). MW, with slightly different pattern, was seen to preserve strongly bound water, while increasing the absorbance of proton hydrates and solvation shells (1364–1387 nm) which may indicate an increase in solvation ability. There was also an increase in the absorbance of water molecules confined between ions (1398 nm).

In the next step, the aquagrams were calculated with both waters’ spectra taken together to try to obtain more general water spectral patterns, irrespective of the water type, and to see what the difference between water spectral patterns is depending on the frequency of the applied sound, and what exactly sound does to water molecular structure independent of sound frequency (Figure 6). The specific WASPs for 432 Hz and 440 Hz were generated when the averaged spectra of controls were subtracted from their corresponding datasets and averaging according to the frequency was performed. 

Even though there were differences at specific water absorbance bands depending on the sound frequency, in general it can be concluded that the effect of sound on water resulted in increased absorbance of strongly bound, ice-like water. In another words, if the differences between the waters are not considered, on average, sounds at 432 Hz and 440 Hz frequency promoted crystallization of water. However, the aquagrams in Figure 6 can be somewhat misleading as they are calculated using averaged spectra of both waters together and may look contradictory to the previously presented aquagrams, especially the ones given in Figure 5b, which display WASPs of waters perturbed by 440 Hz sound. This apparent contradiction is exactly the result of averaging, where the much stronger influence of sound of 432 Hz masked the less influential effects of 440 Hz. This just emphasizes the importance of the finding that the effects of the sound on water molecular network are sample- and frequency-dependent. 

## 3. Discussion

In this study, aquaphotomics methodology was applied to investigate the influence of sound effects on water samples monitored using near infrared spectroscopy. Using the water matrix coordinates (WAMACS) [15,38], as is usual in aquaphotomics, the changes in the molecular network of water samples as a result of sound perturbation were described. The most important, prominent WABs in characterizing the sound effects are summarized in Table 3, where those that appeared consistently during different spectral analyses are marked with darker color and could be considered as WAMACs—absorbance bands of water at which the effect of the sound can be measured.

Repeatedly-present water species in all the conducted analyses for “after sound” samples were indicative of sound perturbation, most consistent of which at the 1300–1600 nm region was the water pentamer (1484–1496 nm), or water molecules bound by four hydrogen bonds [15]. This water molecular species is well-known as temperature-sensitive, where the absorbance at this band increases with the decrease in temperature [39]. The importance of this band for description of the influence of sound agrees with what was observed in measurements of temperature of the samples after the sound perturbation, where the common change for all samples was a decrease in temperature. This is further evidence of the stimulating effect of sound on hydrogen bond making.

Our results showed that despite the small difference of only 8 Hz, perturbation by sound using frequencies of 432 Hz and 440 Hz produced large and consistent differences in water samples as multiple analyses confirmed and established that they are water-dependent. In other words, it was determined that sound affected pure and mineral water in a different way. This has two implications. First, it provides basis for the future use of sound in perturbation spectroscopy to differentiate between the samples, and second, it implies that other water-based systems may be affected in different ways by the sound of the same frequency. When it comes to the effect of sound on water, in general, it was observed that, on average it led to the reduction in the samples’ variability during the measurements and the samples became more stable against environmental influences. This was observed not only in the spectral analysis, but also in the measurements of physico-chemical parameters. This stability can be explained, as it was succinctly presented in aquagrams, by the effect of sound on the molecular network of water which promotes crystallization. In simpler words, the water which is strongly hydrogen bonded is not easily changed, and therefore it is more stable against influences from the environment. The temperature measurements also support this explanation, as the temperature of all the samples was shown to decrease after sound perturbation. However, if the effects of sounds of different frequencies are considered separately, which was shown to be the most correct, the effect of sound with 432 Hz frequency promotes crystallization, and the effects are much stronger compared to the effects of 440 Hz sound, which are actually opposite and could be said to enhance evaporation and solubilization.

Our findings, even though based on investigation of sound effects on the simplest aqueous systems and only two frequencies, may prove to have wider implications, especially considering the major role water plays as a matrix for biological systems. Recent scientific reports showed that audible sound promotes crystallization of proteins, which is frequency-dependent, and shows some variation based on protein type; this was found to be connected with the change of temperature and evaporation of protein solution [6,10]. Although the mentioned studies were performed using different frequencies and even variable-frequency sound perturbation, there is a common link with our study—sound affects water-based systems and changes their molecular structure in a frequency- and sample-type-dependent manner.

In conclusion, this study successfully applied NIR spectroscopy for rapid and non-invasive characterization of sound effects on water, and aquaphotomics inquiry allowed for the interpretation of the molecular dynamics after the applied perturbation, giving a better understanding of the sound-water interaction. Future research efforts will be directed towards exploration of the effects of additional frequencies on specific water systems, including biological.

## 4. Materials and Methods

### 4.1. Experimental Setup

As water samples, purified water (PW) (Organo, Purelite-α, Tokyo, Japan) and Yunosato Gold mineral water (MW) (Yunosato Onsen, Hashimoto, Japan) were used. The content of the mineral water is described in the following table (Table 4):

Both waters were kept in similar containers (plastic bottles and glass vials, depending on the experiment) and conditions (at room temperature). Music tuned separately at 432 Hz and 440 Hz was played by the Japanese pianist and composer Acoon Hibino on a YAMAHA MOTIF XF8 synthesizer, sounded by a pair of BOSE L1 Compact stereo speakers. The PW and MW samples were placed a meter away from the sound source inside glass vials, specifically made for measurements with a MicroNIR spectrophotometer by VIAVI Solutions (Scottsdale, AZ, USA). Each water type was prepared with 2 replicates in a total of 4 vials per frequency, each measured 5 consecutive times. Sample replicates at every frequency were measured in random order, first spectra were taken at 432 Hz, then at 440 Hz, for a total of 80 spectra.

**Figure 7:**
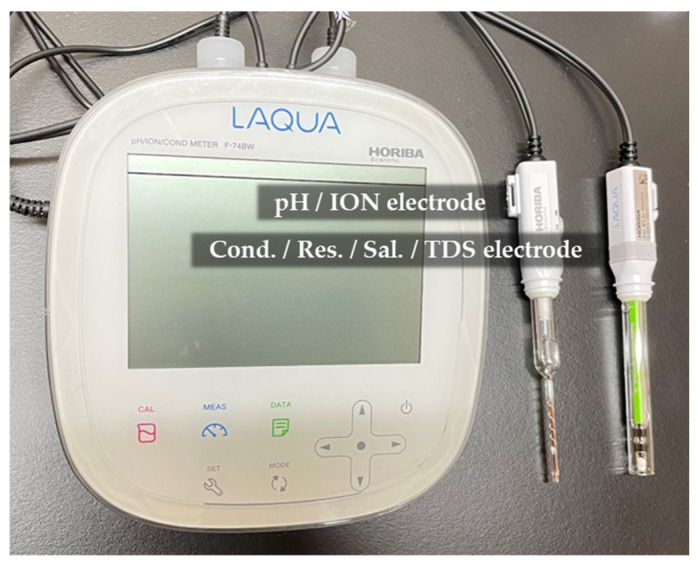
LAQUA Horiba F-74BW meter with pH electrode (left) and electrical conductivity/resistivity/salinity electrode (right).

Several sample parameters were measured before and after sound by LAQUA Horiba F-74BW meter, such as pH, electrical conductivity, electrical resistivity, salinity and sample temperature (Figure 7).

### 4.2. Near-Infrared Spectroscopy

Near-infrared spectroscopy was selected as it is a rapid, non-destructive and non-invasive measurement technique, that requires very little or no sample preparation at all, and it can also be used for real-time monitoring. In the near-infrared spectral region (780–2500 nm) there are four main water absorbance maxima located at around 970 nm, 1190 nm, 1450 nm and 1940 nm, due to the 2nd overtone of the OH stretching band, the combination of the first overtone of the OH stretching and OH bending band, 1st overtone of the OH stretching bands and combination of the OH stretching band and OH bending band, respectively [40]. Following the aquaphotomics findings and systematization of the knowledge about water, these main bands are even further resolved and currently there are more than 500 known absorbance bands in this region [41], which is why this technique is specifically chosen to investigate the molecular structure of water under influence of sound perturbation. 

**Figure 8:**
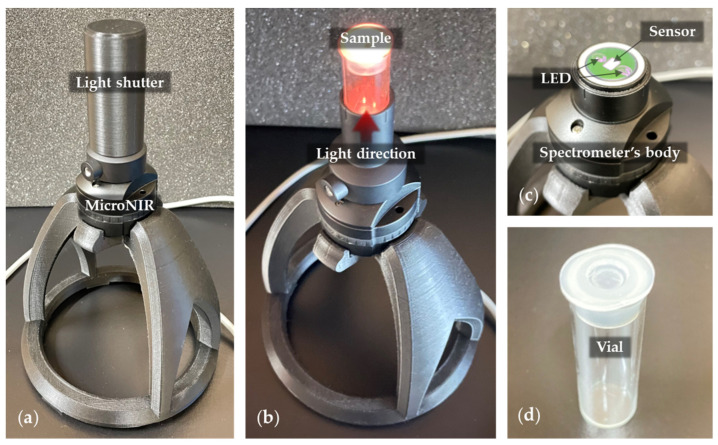
Near-infrared spectroscopy setup: (**a**) MicroNIR 1700-ES mounted on a 3D-printed stand and with a 3D-printed light shutter cap; (**b**) setup without light shutter, showing the sample’s position and measurement; (**c**) MicroNIR 1700-ES device; (**d**) empty glass vial as a sample container, with 2 replicates per water at specific frequency, in a total of 40 spectra acquired (2 waters at 2 frequencies with 2 replicates, each measured 5 consecutive times).

The spectrophotometer used for this study was a MicroNIR 1700-ES (Viavi Solutions, Scottsdale, AZ, USA), capable of acquiring spectra in the wavelength range of 908.1–1676.2 nm, with a wavelength step of approximately 6 nm. The device was set on reflectance mode and used with its vial-holder attachment and a 3D-printed light-shutter cap (Figure 8a). During the experiments, samples were measured before and after the played sounds, where each vial had its spectra taken 5 times consecutively and represented as 1 sample.

**Figure 9:**
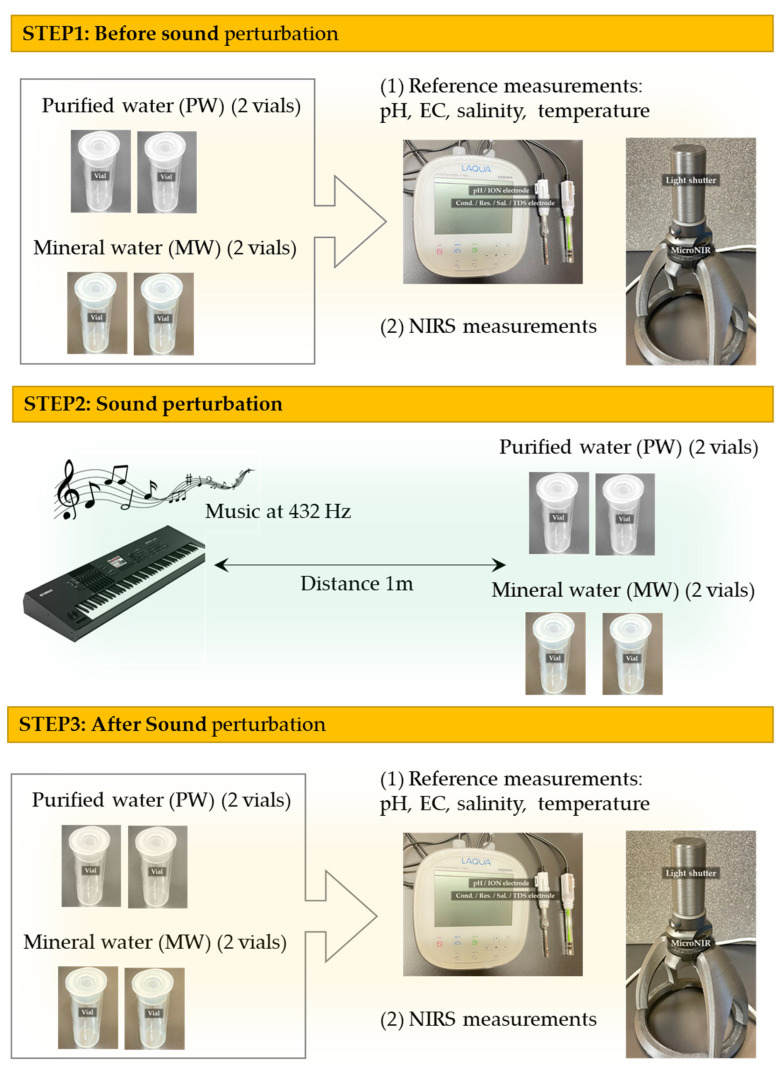
Schematic representation of the experimental flow. The experiment was first performed in steps 1–3 with the frequency of music set to 432 Hz, and then the entire procedure was repeated with the only difference being setting of the frequency to 440 Hz.

The entire experimental flow is given schematically in Figure 9. Steps 1–3 were first performed for the sound perturbation using 432 Hz music, then the entire experiment was repeated, using the 440 Hz frequency.

### 4.3. Aquaphotomics Spectral Analysis

In this study, the main focus of the analysis was placed on the 1st water overtone (1300–1600 nm), where several analytical methods were applied to the acquired spectral data, such as calculation of difference spectra and MVA (multi-variate analysis), including PCA (principal component analysis) [42] and SIMCA (soft independent modeling by class analogy) [43]. This particular wavelength region was chosen since it is by far the best studied in aquaphotomics; there is almost no overlap with the absorbance of other functional groups and it provides the most information about the molecular structure of water [15]. 

Difference spectra were calculated by subtracting the values of a control’s selected wavelength *λ_control_* (subtrahend) from the same wavelength of the sample in focus *λ* (minuend), where the difference is represented as *λ_DSA_*:

(1) *λ_DSA_* = *λ* − *λ_control_*

With this calculation applied to all spectra, fundamental and environmental effects are reduced, being left only with other perturbations that have occurred and are not present equally in all samples (such as sound).

As preprocessing, SNV (standard normal variate) [44] was performed in this and all other analyses to reduce spectral baseline offset. In addition to SNV, the spectral data were smoothed using the Savitzky–Golay 2nd order polynomial filter (9 points) [45].

As an exploratory analysis, PCA (principal component analysis) was used, a statistical procedure that forms the basis of all used MVA (multi-variate analysis). It allows summarizing of information content in large data tables by means of a smaller set of “summary indices” that can be more easily visualized and analyzed; in the form of scores and loading plots in this study. Scores are projections of original spectra in the pattern spaces defined by principal components, while loadings show the weight coefficients of original variables. Mean-centering was applied only in PCA calculations as an additive transformation, after which each sample became relative to the global mean and analyses were of the variance around the global mean. Another statistical method used in this study is SIMCA (soft independent modeling by class analogy), applied for supervised classification of data, with confidence interval at 95%. Classification of spectra is based on a comparison of Mahalanobis distance, which is the distance between the spectrum and the centroid of each class. As an additional transformation, specifically used in recalculating presented SIMCA discriminating powers, feature scaling (unity-based normalization) was applied, a technique that brings all values within the range from 0 to 1, where the smallest value becomes 0, the biggest becomes 1, and all other values are spread in between, calculated by the following formula:

(2) $$X^{\prime }=\frac{X-X_{min}}{X_{max\;}-X_{min}}$$

where X is the selected value, $X^{\prime }$ is the resulting normalized value, $X_{min}$ is the smallest (minimum) and $X_{max}$ is the biggest (maximum) values within each discriminating power table. All MVA analyses were performed using commercially available multivariate analysis software, Pirouette (version 4.5, Infometrix, Bothell, WA, USA).

After conducting the listed MVA above, prominent WABs were chosen for distinguishing the effects from sound and aquagrams were generated for illustrating the spectral patterns of the averaged datasets in a simple manner:

(3) $$A{{}^{\prime }}_{\lambda }=\frac{A_{\lambda }-\mu_{\lambda }}{\sigma_{\lambda }}$$

where *λ* is the selected wavelength, *A′_λ_* is the resulting wavelength value in the aquagram, *A_λ_* is the absorbance after applying SNV preprocessing, *μ_λ_* is the mean for all values at the specific wavelength and *σ_λ_* is the standard deviation of the same wavelength.

Assignments for the main WAMACS of the 1st water overtone are provided in Table 5 and used for characterization and interpretation of the changes in water molecular structure of the tested samples.

## Figures and Tables

**Table 1:** Measured water parameters, each presented with its mean and standard deviation (SD), with increase in value stability for the perturbed waters, compared to the control ones in general also having higher standard deviations. A similar trend in changes was seen through all the samples.

	pH	Electrical Conductivity Mean ± SD (μS/cm)	Salinity Mean ± SD (ppb)	Temperature Mean ± SD (°C)
PW-control Before	7.82 ± 0.38	10.25 ± 5.47	0	24.9 ± 0.3
PW-control After	6.95 ± 0.15	37.79 ± 27.7	0	24.6 ± 0.2
MW-control Before	7.09 ± 0.11	302 ± 2	0.15	25 ± 0.1
MW-control After	6.95 ± 0.14	320.5 ± 4.6	0.15	24.6 ± 0.1
PW-432 Hz Before	7.15 ± 0.18	23.7 ± 2.15	0	24.5 ± 0.1
PW-432 Hz After	6.39 ± 0.1	24.4 ± 2.2	0	24 ± 0.1
PW-440 Hz Before	7.49 ± 0.1	19.1 ± 1.19	0	24.3 ± 0.3
PW-440 Hz After	6.81 ± 0.34	64.1 ± 2.17	0	23.7 ± 0.3
MW-432 Hz Before	7.02 ± 0.33	307 ± 2.64	0.15	24.9 ± 0.3
MW-432 Hz After	6.97 ± 0.27	317 ± 2.37	0.15	24.3 ± 0.2
MW-440 Hz Before	7.04 ± 0.18	314 ± 0.6	0.15	24.8 ± 0.1
MW-440 Hz After	6.81 ± 0.27	319 ± 0.8	0.15	24.3 ± 0.1

**Table 2:** Water absorbance bands (WABs) prominent for distinguishing samples perturbed by sound from the ones used as a control (before sound perturbation). From yellow to red, repeatability of the same band activation increases in the 4 displayed datasets, where most consistent and notable water matrix coordinates (WAMACS) were shown in darker color.

PW-432 Hz	1304			1347	1354	1372	1385	1398	1409	1422	1440	1447	1465		1496		1515	1528		1546	1564		1583
PW-440 Hz	1304	1316	1335	1347		1372		1398	1416		1434	1447	1465	1477	1496	1508				1552	1564		1583
MW-432 Hz	1310		1335	1354	1366	1372	1385	1398	1403	1422	1440		1459	1477	1496		1515			1552	1564		
MW-440 Hz	1304	1316	1329	1347	1360	1372		1391	1409	1422		1453		1477	1490	1502	1515		1533	1552	1564	1577	1589

**Table 3:** WABs seen activated through analyses in the region 1300–1600 nm. From lighter to darker (yellow to red) color, repeatability of the same band increases in the displayed 4 datasets. Consistent common bands were found at 1335–1347 nm, 1391–1403 nm, 1409–1416 nm, 1422–1427 nm, 1447 nm, 1484–1496 nm and 1552–1558 nm for sound perturbation, while specific bands were distinguished according to the frequencies and water types.

PW at 432 Hz																							
**DSA**		1316		1347		1372		1391	1403	1422					1496		1515		1539		1564		1583
**PCA**			1323	1347	1366		1385			1422		1453		1471	1490	1508			1539	1558	1564		1583
**SIMCA**	1304			1347	1354	1372	1385	1398	1409	1422	1440	1447	1465		1496		1515	1528		1546	1564		1583
**PLS-DA**	1310	1323	1335	1354	1360	1378		1398	1416	1428	1434	1447	1459	1477	1490	1502	1515	1521	1533	1552	1564	1577	1595
PW at 440 Hz																							
**DSA**			1329	1354				1398	1409	1428		1447	1465	1477	1490			1521	1539	1558		1577	
**PCA**			1335	1354	1360			1398	1409	1428		1447	1465	1477	1490			1521		1558		1577	
**SIMCA**	1304	1316	1335	1347		1372		1398	1416		1434	1447	1465	1477	1496	1508				1552	1564		1583
**PLS-DA**	1304	1323	1335	1354		1372	1385	1398	1416	1422	1447	1453		1471	1490	1508	1521	1539	1546	1558		1570	1595
MW at 432 Hz																							
**DSA**			1323	1347		1378		1391			1434	1447	1459		1496		1515			1558		1577	
**PCA**		1316	1335	1347		1372	1385		1409		1434	1447	1459		1484	1508			1539	1558		1577	
**SIMCA**	1310		1335	1354	1366	1372	1385	1398	1403	1422	1440		1459	1477	1496		1515			1552	1564		
**PLS-DA**	1310	1323	1335	1347	1366	1378		1398	1416		1434	1447	1465	1477	1490	1508		1533	1546	1558	1564	1577	1583
MW at 440 Hz																							
**DSA**			1329		1360			1391	1403	1428		1447	1465		1490	1502	1515	1527	1539	1558		1577	
**PCA**				1347	1360			1391	1403	1428		1447	1465		1484	1502	1521	1527	1539	1558		1577	
**SIMCA**	1304	1316	1329	1347	1360	1372		1391	1409	1422		1453		1477	1490	1502	1515		1533	1552	1564	1577	1589
**PLS-DA**	1304	1323	1341	1347	1360	1378		1398	1416	1428	1440	1447		1471	1484	1502		1528	1539	1552	1564	1570	1583

**Table 4:** Mineral content for Yunosato Gold mineral water (MW) as described on the bottle’s label.

Nutritional Information Per 1000 mL	
Calories from proteins, fats, carbohydrates	0 mg
Na	40 mg
Ca	23 mg
Mg	8.7 mg
K	2.2 mg

**Table 5:** Water matrix coordinates (WAMACS) in the 1st water overtone and their assignments. All assignments are based on Tsenkova 2009 [15], unless otherwise indicated.

WAMACS	Assignment	Significance/Phenomena in Biological and Aqueous Systems for Which the WAMACS Was Found Important
C1: 1336–1348 nm	ν3, H_2_O asymmetric stretching vibration, proton hydration [28]	Self-organization [29], water activity [46], germination [47].
C2: 1360–1366 nm	Water solvation shell, OH-(H_2_O)_1,2,4_, Ion hydration, proton hydration [46]	Water vapor/moisture absorbance bands [41,46], self-organization [29], water activity [46], viability [48], germination [47], firmness [49], hardness [50], solubility [29]
C3: 1370–1376 nm	ν1 + ν3, symmetric and asymmetric stretching vibration Ion hydration, proton hydration [46]	
C4: 1380–1388 nm	Water solvation shell, OH-(H_2_O)_1,4_ and/or superoxide- tetrahydrate, O_2_-(H_2_O)_4_ Ion hydration, proton hydration [46]	
C5: 1398–1418 nm	Water confined in the local field of ions (1396–1403 nm) [32] 1st overtone of the free OH group trapped in the hydrophobic interior [51]	Water activity [46], drying and dehydration [52], expulsion of cellular water, damage [53,54]
	Free water molecules and free OH-(S0)	Moisture content [55,56], water activity [46], seed vitality [57]
C6: 1421–1430 nm	Water hydration, H-OH bend and O…O	Protein hydration, protein fibrillation [58,59], water activity [46,60], damage and defects [61,62,63], amorphous phase [64,65,66,67,68,69], density [65]
C7: 1432–1444 nm	Water molecules with 1 hydrogen bond (S1)	Phase transition, sugar-water interaction [70,71], hardness [50], seed vitality [55], protection against dehydration [72]
C8: 1448–1454 nm	ν2 + ν3, Water solvation shell, OH-(H_2_O)_4,5_ Bulk water [46]	Water activity [46,60], viral infection in plants [73], damage [53]
C9: 1458–1468 nm	Water molecules with 2 hydrogen bonds (S2)	Water-protein interaction [58,74,75], firmness [49],
C10: 1472–1482 nm	Water molecules with 3 hydrogen bonds (S3)	Semi-crystalline phase [64,65,66,67,68,69], firmness [49]
C11: 1482–1495 nm	Water molecules with 4 hydrogen bonds (S4)	Damage/preservation (1496 nm) [53,72], firmness [49]
C12: 1506–1516 nm	ν1, ν2, symmetrical stretching, strongly bound water	Structural water, preservation/damage [53,61,76,77], seed viability [48,78]

## Data Availability

The data presented in this study are available on request from the corresponding author. The data are currently not publicly available due to the organization of the local repository that will be open to public in future.

## Sample Availability

Samples of the waters are available from the corresponding authors on reasonable request.
